# Frequencies of circulating myeloid derived suppressor cells and dendritic cells in Egyptian patients with chronic Hepatitis C Virus infection undergoing treatment with IFN-α-based therapy

**DOI:** 10.1186/2051-1426-1-S1-P248

**Published:** 2013-11-07

**Authors:** Mohamed L Salem, Abdel-Aziz A Zidan, Mohamed Abou Senna, Abdel Raouf Abou Al-Azm, Hasan Albatei, Maha Aldemelaawy, Mohamed Attia

**Affiliations:** 1 Faculty of Science, Tanta University, Tanta, Egypt; 2 Biomedical Technology Department, City of Scientific Research and Applied Technologies, Alexandria, Egypt

## Background

Hepatitis C Virus (HCV) is epidemic in Egypt and causes chronic hepatitis. Anti-HCVtherapy (IFN-α and Ribavirin) is only effective in 60% of patients with chronic HCV infection. This failure which is often associates with suppression of immunity results in progression of the disease and the development of hepatocellular carcinoma. Recent studies including ours have shown a positive correlation between accumulation of myeloid derived suppressor cells (MDSCs) and suppressed immunity in cancer and other diseases.

## Aim

To assess the frequency of myeloid cells, including MDCS and dendritic cells (DCs) in chronic HCV patients and correlate it with the responses of the patients to IFN-α-bases therapy.

## Methods

Peripheral blood was drawn from 80 patients with chronic HCV infection (mean age = 41.5 ± 6.51 years; male/female: 60/20) and from10 healthy volunteers (mean age = 28.5 ± 3.81 years; male/female: 8/2). The study was conducted from January 2011 to April 2013. The patients were categorized into responders and non-responders based on viral titer and the clinical data was collected and analyzed for each patient. Frequency of the cells was assessed by flow cytometry and IL-2 was assessed by ELISA.

## Results

We defined MDSC population as Lin-/HLA-DR-/CD33+/CD11b+. We also found increases in the frequency of MDSC. The high levels of MDSC was associated with increases in the frequency of DCs and T cells (CD4+ and CD8+), as well as with the differential count of lymphocytes, and monocytes. It was associated, however, with decreases in the total numbers of the total number of white blood cells, granulocytes, and platelets. The total Bilirubin level, and hemoglobin, in all IFN-α Responders and Non-Responders when compared with healthy donors. Interestingly, the frequencies of MDSC and DCs in IFN-α-responders were lower than in those in non-responders. More interestingly, the levels of MDSC measured 4-6 months of IFN-α treatment of responders was much lower than those during treatment. We found that there was no correlation between MDSCs, and the liver enzymes AST and ALT.

## Conclusions

Chronic HCV patients showed high levels of MDSCs regardless IFN-α therapy. The responders have the tendency of lower MDSC levels than non-responders. MDSCs can use as biomarker of responsiveness to IFN-based therapy. As such, identifying novel effective therapeutic that can target MDSCs would improve clinical outcomes in HCV patients.

